# Effect of Tight Junction-Modulating FCIGRL-Modified Peptides on the Intestinal Absorption of Doxorubicin in Rats

**DOI:** 10.3390/pharmaceutics16050650

**Published:** 2024-05-11

**Authors:** Keon-Hyoung Song

**Affiliations:** Department of Pharmaceutical Engineering, College of Medical Sciences, Soonchunhyang University, Asan 31538, Republic of Korea; beophyen@sch.ac.kr

**Keywords:** doxorubicin, absorption enhancer, tight junction, drug delivery

## Abstract

Doxorubicin is a potent chemotherapy drug, but its oral bioavailability is limited due to its low membrane permeability. Thus, absorption enhancers such as zonula occludens toxin and its six-mer fragment, FCIGRL, have been studied to address this issue. This study aimed to evaluate the effectiveness of four peptides (Pep1, Pep2, Pep3, and Pep4) derived from FCIGRL and investigate the changes in the absorption of doxorubicin, to propose an absorption enhancer for doxorubicin. Pep1 is a modified version of FCIGRL in which the hydroxyl group at the C-terminus is replaced with an amino group. Pep2 is a modified Pep1 in which cysteine is replaced with N_3_-substituted dipropionic acid. Pep3 and Pep4 are Pep2-modified homodimers. Pharmacokinetic analysis was performed in rats after the intraduodenal administration of doxorubicin solutions containing each FCIGRL-modified peptide and the stabilizer levan or benzalkonium chloride (BC). The results showed that Pep3 and Pep4 administered with levan each significantly increased the intestinal absorption of doxorubicin, as did Pep2 administered with levan/BC. In particular, 10 mg·kg^−1^ of Pep4 with levan significantly increased the area under the curve (AUC)_0–240min_ of doxorubicin by 2.38-fold (*p* < 0.01) and the peak concentration (C_max_) by 3.30-fold (*p* < 0.01) compared to the control solution. The study findings indicate that Pep2, Pep3, and primarily Pep4 are novel absorption enhancers that can open tight junctions for doxorubicin, and the effectiveness of the peptides was directly affected by the presence of levan or levan/BC.

## 1. Introduction

Doxorubicin is a potent chemotherapy drug that is used to treat various cancers, such as solid tumors, leukemias, and lymphomas [1]. However, its administration is associated with certain issues. The intravenous administration of doxorubicin, which is commonly used, can cause systemic toxicity [2]. Although oral administration is preferred for its ease of use and patient compliance, increasing doxorubicin bioavailability is difficult [2,3]. Doxorubicin, a biopharmaceutics classification system class III drug, has low oral bioavailability due to its inherently low permeability and because it is a substrate of both P-glycoprotein (P-gp) and cytochrome P450 (CYP) [4,5]. A recent study revealed that the limited and paracellular intestinal absorption of doxorubicin is the major factor responsible for its low oral bioavailability, and P-gp-mediated efflux and CYP-mediated metabolism are not significant barriers to the absorptive transport of doxorubicin [6]. Thus, one formulation strategy that can be employed to enhance the intercellular permeability of doxorubicin and improve its bioavailability is the use of an appropriate absorption enhancer [7].

The absorption of hydrophilic drugs, such as doxorubicin, is substantially limited to the paracellular pathway, where permeation is regulated by tight junctions that join adjacent cells in the absence of an active transport process [8]. Tight junctions are dynamic structures that can be modulated by endogenous and exogenous stimuli, and their reversible opening by controlled modulation provides a potentially safe and effective strategy for enhancing drug absorption [9]. Zonula occludens toxin (ZOT) is an endotoxin that is produced by *Vibrio cholerae* and consists of a single polypeptide chain of 399 amino acids [10,11]. Studies have shown that ZOT binds to a specific receptor on the surface of epithelial cells and can reversibly modulate tight junctions, enhancing the absorption of several hydrophilic and macromolecular drugs with low bioavailability [12,13,14,15]. In particular, the transport of doxorubicin was increased by ZOT in Caco-2 cells and bovine microvascular endothelial cells [16,17]. Further studies have focused on the size truncation of ZOT and found that FCIGRL, a six-mer fragment of the 288–293 amino acid residues of ZOT, retained the absorption-enhancing effect of ZOT on the permeability of poorly absorbable drugs in a non-toxic manner [18,19].

Thus, FCIGRL could be considered a promising absorption enhancer because of its small molecular weight, reversible opening of tight junctions, and non-cytotoxicity. Additional studies suggested that the amidation of the C-terminal amino acid could increase the stability of FCIGRL and further improve its permeation-enhancing efficacy [20,21]. A study on the structure–activity relationship indicated that a stable and more potent analog of FCIGRL could be produced by substituting the cysteine residue at position 2 of FCIGRL [22]. In addition, levan, a fructose polymer, and benzalkonium chloride (BC) have been reported to contribute to the stabilization of peptides [21]. Therefore, this study hypothesized that cysteine modifications of FCIRGL and the co-administration of levan or BC would further strengthen the permeation-enhancing efficacy of FCIRGL, increasing the paracellular absorption of doxorubicin.

The aim of this study was to evaluate the effectiveness of FCIGRL-modified peptides as absorption enhancers and determine changes in the absorption of doxorubicin. Thus, this study performed a pharmacokinetic analysis after the intraduodenal co-administration of doxorubicin and FCIGRL-modified peptides with levan or BC to rats.

## 2. Materials and Methods

### 2.1. Materials

Peptides (>95%) FCIGRL-NH_2_ (Pep1), FXIGRL-NH_2_ (X = N_3_-substituted dipropionic acid; Pep2), the cysteine homodimer of FCIGRL-NH_2_ (Pep3), and the cysteine homodimer of XFCIGRL-NH_2_ (X = N_3_-substituted dipropionic acid; Pep4) were purchased from AnyGen Co., Ltd. (Gwangju, Republic of Korea), and stored at −70 °C before use. AnyGen Co., Ltd. provided a certificate of analysis for each peptide. Doxorubicin, daunorubicin, benzalkonium chloride (BC), and trifluoroacetic acid (TFA) were purchased from Sigma Chemical Co. (St. Louis, MO, USA). Levan was donated by RealBioTech Co., Ltd. (Gongju, Republic of Korea). Acetonitrile, methanol, and ethanol were of high-performance liquid chromatography (HPLC) grade (Fisher Scientific; Fair Lawn, NJ, USA). All solutions were prepared with HPLC-grade water purified by an Arium Mini Plus system (H20-MA-T, Sartorius, Gottingen, Germany) and cellulose nitrate membrane filters (47 mm, 0.2 µm, Whatman, Maidstone, UK). Zoletil 50 for injection was purchased from Virbac (Carros, France) with permission from the Korea Food and Drug Administration. Polyethylene tubing (O.D., 0.965 mm; PE-50) was obtained from Becton Dickinson (Sparks, MD, USA). All surgical supplies were purchased from Professional Hospital Furnishers (Punjab, Pakistan). All other reagents were of analytical grade or better.

### 2.2. Animals

Male Sprague–Dawley rats weighing 280–290 g were purchased from Koatech Corp. (Pyeongtaek, Republic of Korea). The rats were housed individually in cages and allowed to acclimate for at least 2 days after arrival. They were fed standard rat chow and water ad libitum and maintained on a 12 h light/dark cycle. Before the study, the rats were fasted overnight with free access to water. The animal study protocol was approved by the Soonchunhyang University Institutional Animal Care and Use Committee (No. SCH21-0007).

### 2.3. Preparation of Doxorubicin Solutions for the Animal Study

A solution of doxorubicin alone was prepared by sequentially mixing doxorubicin with ethanol and filtered water. Doxorubicin solutions with levan and BC were made by adding levan solution or levan/BC solution to doxorubicin solution. The concentrations of doxorubicin and ethanol were adjusted to ensure that the final concentrations of doxorubicin and ethanol were the same in all solutions (2.5 mg·mL^−1^ of doxorubicin, 10 *v*/*v*% ethanol, 0.25 *w*/*v*% or 0.5 *w*/*v*% of levan, and 0.5 *w*/*v*% BC). Doxorubicin solutions containing each peptide and additive were prepared by adding an appropriate amount of the peptide to the doxorubicin/additive solutions so that the peptide concentration was either 2.5 mg·mL^−1^ or 5 mg·mL^−1^. All doxorubicin solutions were prepared immediately prior to administration based on doses determined in a preliminary study. The following doxorubicin solutions were prepared for administration to the rats: doxorubicin alone (*n* = 3), doxorubicin/levan (*n* = 5), doxorubicin/levan/Pep1 (*n* = 3), doxorubicin/levan/Pep2 (*n* = 3), doxorubicin/levan/Pep3 (*n* = 3 for the 0.25 *w*/*v*% levan group and *n* = 5 for the 0.5 *w*/*v*% levan group), doxorubicin/levan/Pep4 (*n* = 5 for the 0.25 *w*/*v*% levan group and *n* = 3 for the 0.5 *w*/*v*% levan group), doxorubicin/levan/BC (*n* = 3), and doxorubicin/levan/BC/Pep2 (*n* = 5 for the 2.5 mg·mL^−1^ Pep2 group and *n* = 4 for the 5 mg·mL^−1^ Pep2 group).

### 2.4. Intraduodenal Administration of Doxorubicin Solutions to Rats

The rats were anesthetized with an intramuscular injection of Zoletil 50 (25 mg·kg^−1^) prior to the intraduodenal administration of the doxorubicin solutions. The femoral vein and artery were cannulated with PE-50 before the solutions were slowly administered into the duodenum of the cannulated rats at a volume dose of 2 mL·kg^−1^ rat. After administration, the incisions in the duodenum and abdomen were closed. Blood samples of 250 µL were taken 5, 10, 20, 40, 60, 120, 180, and 240 min after administration via the femoral cannula into polypropylene tubes and centrifuged immediately (18,000× *g* for 15 min) to obtain plasma (100 µL).

Deproteinization of the plasma and the addition of an internal standard (IS) were performed by adding 500 mL of methanol, including daunorubicin as the IS, to each tube, followed by vortexing for 30 s and centrifuging at 18,000× *g* for 15 min again. The supernatants were then concentrated, and the reconstituted solutions were analyzed using an HPLC-fluorescence system to monitor temporal plasma concentration profiles over 240 min.

### 2.5. HPLC-Fluorescence Conditions

The HPLC system (Shimadzu Corp., Kyoto, Japan) comprised a binary pump (LC-20AD), autosampler (SIL-20A), column oven (CTO-20A), and degasser (DGU-20A). Aliquots (10 µL) of the reconstituted solutions were injected into the HPLC column. Doxorubicin and IS were separated on a C_18_ reversed-phase column (4.6 mm × 250 mm, 5 μm particle size; YMC Co., Ltd., Kyoto, Japan) using gradient elution at a flow rate of 1.0 mL·min^−1^ for 17 min. The mobile phases consisted of acetonitrile containing 0.1% TFA (eluent A) and water containing 0.1% TFA (eluent B). Eluent A was increased from 30% to 50% over 9 min in the gradient program. The column oven temperature was maintained at 40 °C. The outlet from the HPLC column was directly connected to a fluorescence detector (RF-20A; Shimadzu Corp., Kyoto, Japan). The excitation and emission wavelengths of the fluorescence detector were set at 487 and 555 nm, respectively.

### 2.6. Data Analysis

The peak area of doxorubicin in the chromatograms was converted to a concentration using the ratio of the peak area of doxorubicin to the IS peak area in serial dilutions of the stock solution. The pharmacokinetic parameters were calculated using the non-compartmental analysis WinNonlin^®^ pharmacokinetic software package (version 5.3) (Pharsight, Mountain View, CA, USA). The area under the curve (AUC) was calculated using the linear trapezoidal method. The peak concentration (C_max_) and time to reach the peak (T_max_) following administration were determined from the observed data. The results are presented as the mean and standard error of the mean (mean ± SEM). The statistical significance of differences between the solutions and controls was evaluated using Student’s *t*-test and one-way analysis of variance (ANOVA) (SPSS for Windows ver. 12.0, SPSS, Chicago, IL, USA). The significance level was set at *p* < 0.05 or *p* < 0.01.

## 3. Results

### 3.1. Preparation of FCIGRL-Modified Peptides

The FCIGRL-modified peptides were altered either at the C-terminus or cysteine moiety to strengthen the permeation-enhancing efficacy of FCIRGL. Pep1 is a modified version in which the hydroxyl group at the C-terminus of FCIGRL is substituted with an amino group. Pep2 is a modified Pep1 in which cysteine is replaced with N_3_-substituted dipropionic acid. Pep3 is a homodimer that is modified by the disulfide bonding of two Pep2s. Pep4 has two N_3_-substituted dipropionic acid groups added to each of the two N-termini of Pep3.

### 3.2. Intraduodenal Administration of Doxorubicin with FCIGRL-Modified Peptides to Rats

Doxorubicin solutions containing levan, with or without FCIGRL-modified peptides, were prepared to determine the effectiveness of FCIGRL-modified peptides as absorption enhancers for doxorubicin. Male Sprague–Dawley rats cannulated in the femoral vein and artery were randomly assigned to receive one of the following solutions via intraduodenal administration: doxorubicin with levan as a control, doxorubicin with levan and Pep1, doxorubicin with levan and Pep2, doxorubicin with levan and Pep3, and doxorubicin with levan and Pep4. The administered doses were 5 mg·kg^−1^ of doxorubicin, 0.5 *w*/*v*% levan, and 10 mg·kg^−1^ of each peptide.

**Figure 1:**
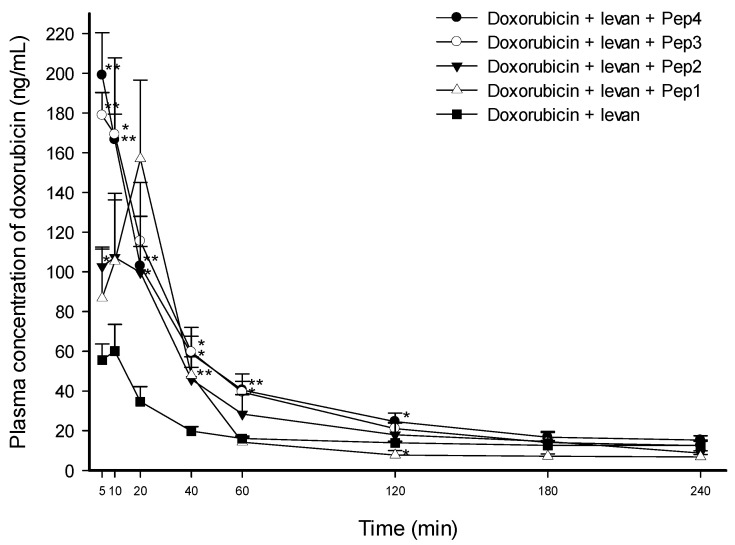
Average plasma concentrations of doxorubicin over time in Sprague–Dawley rats after the intraduodenal administration of doxorubicin solutions containing FCIGRL-modified peptides and levan (5 mg·kg^−1^ of doxorubicin and 10 mg·kg^−1^ of each FCIGRL-modified peptide in 0.5 *w*/*v*% levan solution). Each data point represents the mean ± standard error of the mean (SEM) of 3–5 rats. Significant differences at * *p* < 0.05 and ** *p* < 0.01 compared to the doxorubicin/levan control solution at each time point.

Figure 1 shows the mean (±SEM) plasma concentration of doxorubicin versus time profiles after the administration of the doxorubicin solutions. The plasma levels of doxorubicin were significantly and statistically higher for all doxorubicin solutions containing levan and each FCIGRL-modified peptide than for the control solution of doxorubicin/levan at different time points. In particular, the plasma concentration of doxorubicin in the Pep1 solution was 2.41 times (*p* < 0.01) higher at 40 min. The Pep2 solution showed 1.85-fold (*p* < 0.05) and 2.88-fold (*p* < 0.05) higher concentrations of doxorubicin at 5 min and 20 min, respectively. The Pep3 solution showed 3.21-fold (*p* < 0.01), 2.81-fold (*p* < 0.05), 3.01-fold (*p* < 0.05), and 2.45-fold (*p* < 0.05) higher concentrations of doxorubicin at 5 min, 10 min, 40 min, and 60 min, respectively. The Pep4 solution showed 3.58-fold (*p* < 0.01), 2.77-fold (*p* < 0.01), 2.97-fold (*p* < 0.01), 2.97-fold (*p* < 0.05), 2.52-fold (*p* < 0.01), and 1.77-fold (*p* < 0.05) higher concentrations of doxorubicin at 5 min, 10 min, 20 min, 40 min, 60 min, and 120 min, respectively, compared to the control solution of doxorubicin/levan.

Correspondingly, the pharmacokinetic parameters calculated after administering the doxorubicin solutions showed differences between the doxorubicin/levan/peptide solutions and the control doxorubicin/levan solution (Table 1). The C_max_ of Pep1, Pep2, Pep3, and Pep4 with levan increased by 2.34-fold (*p* < 0.05), 1.97-fold (*p* < 0.05), 3.07-fold (*p* < 0.01), and 2.97-fold (*p* < 0.01), respectively, compared to the control solution. The AUC_0–240min_ of Pep3 and Pep4 with levan was enhanced by 2.15-fold (*p* < 0.01) and 2.24-fold (*p* < 0.05), respectively, compared to the control solution. However, the AUC_0–240min_ of Pep1 and Pep2 only increased by 1.46-fold and 1.66-fold, respectively, with no statistical differences compared to the control solution.

These results indicate that only Pep3 and Pep4 solutions containing levan significantly and statistically enhanced the intestinal absorption of doxorubicin.

### 3.3. Effect of Pep3 and Pep4 with Levan on the Permeability of Doxorubicin

Among the FCIGRL-modified peptides, the extent and rate of doxorubicin absorption were significantly increased by Pep3 and Pep4 in the presence of added levan. The following solutions were prepared and administered intraduodenally to the rats to evaluate the impact of Pep3, Pep4, and levan concentrations on the absorption of doxorubicin (5 mg·kg^−1^): doxorubicin alone, doxorubicin with levan (0.25 or 0.5 *w*/*v*%) and Pep3 (5 or 10 mg·kg^−1^), doxorubicin with levan (0.25 *w*/*v*%) and Pep4 (10 mg·kg^−1^), and doxorubicin with levan (0.5 *w*/*v*%) and Pep4 (5 or 10 mg·kg^−1^). Doxorubicin alone was used as the control to compare differences based on peptide and levan concentrations.

**Figure 2:**
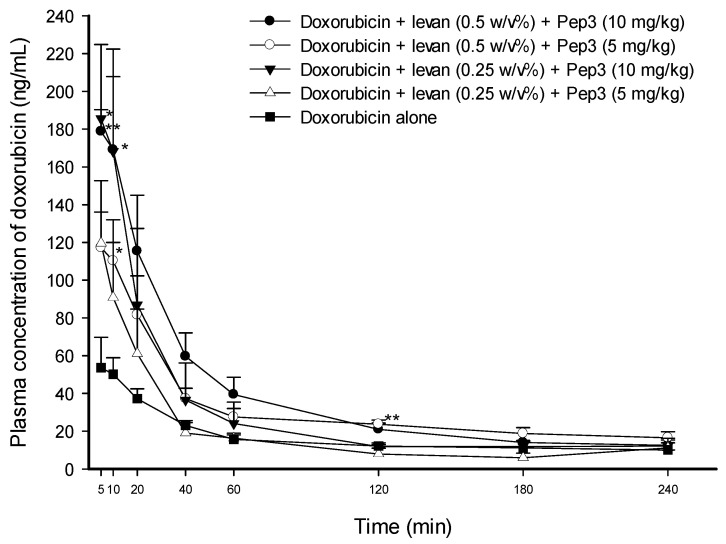
Average plasma concentration–time profiles of doxorubicin in rats after the intraduodenal administration of doxorubicin solutions with different concentrations of levan and Pep3. The dose of doxorubicin was 5 mg·kg^−1^. Each data point represents the mean ± SEM of 3–5 rats. * *p* < 0.05 and ** *p* < 0.01 compared with the control solution of doxorubicin alone.

The administration of the solution containing 5 mg·kg^−1^ of doxorubicin, 0.5 *w*/*v*% levan, and 10 mg·kg^−1^ of Pep3 significantly increased doxorubicin concentrations in the plasma by 3.33 times (*p* < 0.01) at 5 min and 3.37 times (*p* < 0.05) at 10 min compared to the control solution of doxorubicin alone. However, when the solution containing levan and Pep3 was administered with half the concentration of Pep3, the plasma concentration of doxorubicin was increased only 2.20-fold (*p* < 0.05) at 10 min, indicating a decrease in enhancement. Reducing the concentration of levan by half in the solution containing levan and Pep3 led to an increase of 3.46-fold (*p* < 0.05) at 5 min with no statistical difference at other times. When both concentrations of Pep3 and levan were reduced by half, no statistical difference in the plasma concentration of doxorubicin was observed at any time point compared to doxorubicin alone (Figure 2). The pharmacokinetic analysis revealed that the administration of 0.5 *w*/*v*% levan and 10 mg·kg^−1^ of Pep3 produced a statistically significant increase in the AUC_0–240min_ and the C_max_ of 2.29-fold (*p* < 0.05) and 3.41-fold (*p* < 0.01), respectively, compared to doxorubicin alone. Similar to the results seen by decreasing levan and Pep3, the administration of 0.5 *w*/*v*% levan and 5 mg·kg^−1^ of Pep3 increased the AUC_0–240min_ by 1.89-fold (*p* < 0.05) and the C_max_ by 2.07-fold (*p* < 0.05). However, enhancement was reduced compared to the administration of 0.5 *w*/*v*% levan and 10 mg·kg^−1^ of Pep3. The AUC_0–240min_ and the C_max_ of the solutions containing 0.25 *w*/*v*% levan and Pep3 (5 mg·kg^−1^ or 10 mg·kg^−1^) were also not statistically different compared to the control solution of doxorubicin alone (Table 2).

**Figure 3:**
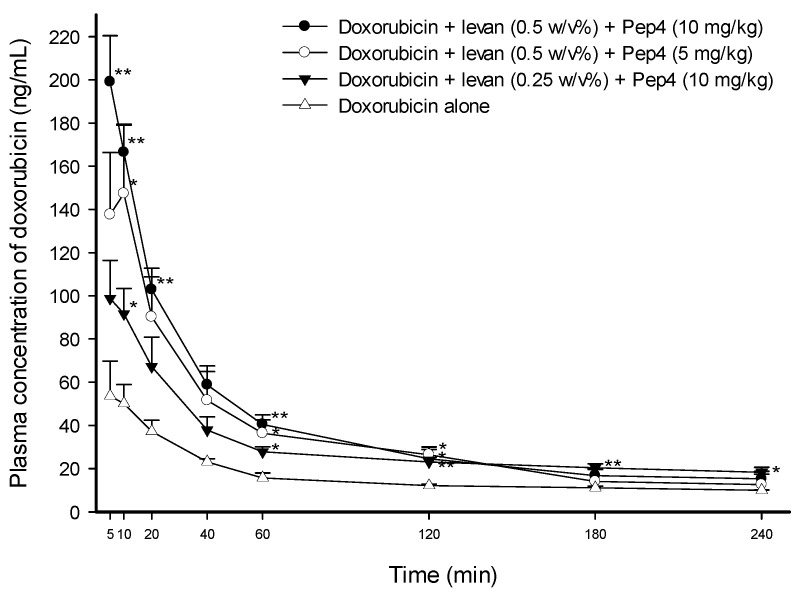
Temporal profiles of doxorubicin concentrations in plasma after the intraduodenal administration of doxorubicin solutions with different concentrations of levan and Pep4. The dose of doxorubicin was 5 mg·kg^−1^. Each data point represents the mean ± SEM of 3–5 rats. * *p* < 0.05 and ** *p* < 0.01 compared with the control solution of doxorubicin alone.

In the study of Pep4, the greatest increases in plasma doxorubicin concentrations were produced by the administration of a solution containing 0.5 *w*/*v*% levan and 10 mg·kg^−1^ of Pep4, with a 3.71-fold increase at 5 min (*p* < 0.01), 3.32-fold at 10 min (*p* < 0.01), and 2.76-fold at 20 min (*p* < 0.01). In comparison, the solution containing 0.5 *w*/*v*% levan and 5 mg·kg^−1^ of Pep4 showed a 2.94-fold increase (*p* < 0.05), and the solution containing 0.25 *w*/*v*% levan and 10 mg·kg^−1^ of Pep4 showed a 1.83-fold increase (*p* < 0.05) at 10 min (Figure 3). The solution containing 0.5 *w*/*v*% levan and 10 mg·kg^−1^ of Pep4 produced the greatest increase in the AUC_0–240min_ by 2.38-fold (*p* < 0.01) and the C_max_ by 3.30-fold (*p* < 0.01) compared to the control solution of doxorubicin alone. The solution with 0.5 *w*/*v*% levan and 5 mg·kg^−1^ of Pep4 increased the AUC_0–240min_ and the C_max_ by 2.11-fold and 2.44-fold, respectively, while the solution with 0.25 *w*/*v*% levan and 10 mg·kg^−1^ of Pep4 showed a 1.81-fold increase in the AUC_0–240min_ (*p* < 0.01) and a 1.72-fold increase in the C_max_ (Table 2).

Overall, the results demonstrated that the absorption of doxorubicin was increased proportionally to the concentration of each peptide and that levan increased the permeation-enhancing efficacy of Pep3 and Pep4.

### 3.4. Effect of Pep2 with Levan and BC on the Permeability of Doxorubicin

As shown in Table 1, Pep2 did not statistically increase the AUC_0–240min_ of doxorubicin in the presence of levan. Assuming that this result was due to a Pep2 stability issue, the intrinsic efficacy of Pep2 was evaluated by adding BC along with levan. The following solutions were prepared to confirm whether the levan and BC additives themselves enhanced the permeability of doxorubicin (5 mg·kg^−1^): doxorubicin alone as a control, doxorubicin with levan (0.5 *w*/*v*%), doxorubicin with levan (0.5 *w*/*v*%) and Pep2 (5 or 10 mg·kg^−1^), doxorubicin with levan (0.5 *w*/*v*%) and BC (0.5 *w*/*v*%), and doxorubicin with levan (0.5 *w*/*v*%), BC (0.5 *w*/*v*%), and Pep2 (5 or 10 mg·kg^−1^).

**Figure 4:**
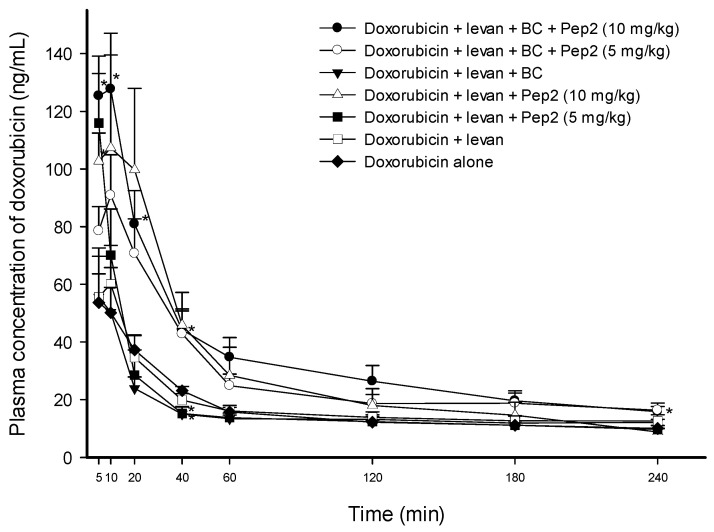
Average plasma concentration versus time profile for doxorubicin in rats after the intraduodenal administration of doxorubicin solutions with different concentrations of Pep2 in the presence of 0.5 *w*/*v*% levan or 0.5 *w*/*v*% BC. The dose of doxorubicin was 5 mg·kg^−1^. Each data point represents the mean ± SEM of 3–5 rats. * Significant difference at *p* < 0.05 compared with the control solution of doxorubicin alone.

Figure 4 shows that no doxorubicin solution containing levan, levan/BC, or levan/Pep2 (5 mg·kg^−1^ or 10 mg·kg^−1^ of Pep2) changed the plasma concentration of doxorubicin at any time point compared to the control solution of doxorubicin alone. Additionally, there was no difference in the AUC_0–240min_ or C_max_ of doxorubicin with the control solution, indicating that the absorption of doxorubicin was unaffected by levan, levan/BC, or Pep2/levan (Table 2). However, when BC was introduced as an additive to the doxorubicin/levan/Pep2 solution, the AUC_0–240min_ of doxorubicin increased by 2.08-fold (*p* < 0.05) and the C_max_ by 2.22-fold (*p* < 0.05), and the plasma concentrations of doxorubicin were enhanced by 2.34-fold at 5 min, 2.55-fold at 10 min, 2.17-fold at 20 min, 1.91-fold at 40 min, and 1.58-fold at 240 min (*p* < 0.05). Even 5 mg·kg^−1^ of Pep2 increased the AUC_0–240min_ by 1.68-fold (*p* < 0.05) compared to doxorubicin alone (Table 2).

Thus, the permeability of doxorubicin was enhanced by Pep2 in the presence of both levan and BC. The results also suggest that FCIGRL-modified peptides require suitable additives to achieve their intrinsic efficacy.

## 4. Discussion

Low bioavailability is a major challenge in drug development that can affect drug effectiveness. One approach to overcoming this problem is to use an absorption enhancer, which can increase drug permeation through a paracellular pathway, reduce the dosage, and extend the drug’s therapeutic benefits. Ideal absorption enhancers should quickly enhance drug absorption, have a reversible effect, and be non-toxic to the cellular membrane or tight junction cytoskeletons at the practical concentration level.

ZOT, the endotoxin produced by *Vibrio cholerae*, can modulate tight junctions and exhibit non-cytotoxic properties, thereby improving the absorption of various hydrophilic and macromolecular drugs with poor bioavailability [16,17]. FCIGRL, the six-mer fragment of ZOT, was reported to maintain the permeation-enhancing efficacy of ZOT without causing cytotoxicity [18,19]. For instance, in Caco-2 cell studies, a ZOT concentration of 4.0 μg·mL^−1^ enhanced the apparent permeability coefficient (*P_app_*) of PEG4000 and inulin by 1.17-fold (*p* < 0.05) and 6.24-fold (*p* < 0.05), respectively [16]. FCIGRL increased the AUC_0–360min_ values of PEG4000 and inulin by 2.35-fold (*p* < 0.05) and 2.92-fold (*p* < 0.05) compared to each control of PEG4000 alone and inulin alone when administered intranasally with 10 mg·kg^−1^ of FCIGRL to rats [19]. Similar to other drugs, ZOT increased the *P_app_* of doxorubicin by 2.29-fold (*p* < 0.05) and 2.64-fold (*p* < 0.05) in Caco-2 transport studies [16] and by 1.46-fold (*p* < 0.05) and 1.86-fold (*p* < 0.05) across bovine brain microvessel endothelial cell monolayers [17] at concentrations of 2.0 μg·mL^−1^ and 4.0 μg·mL^−1^, respectively.

This study was conducted to assess whether four peptides derived from the ZOT fraction (i.e., Pep1, Pep2, Pep3, and Pep4) maintained the efficacy of ZOT and enhanced the intestinal absorption of doxorubicin. However, in a preliminary study, no statistical difference in the AUC of doxorubicin was observed after intraduodenal administrations of doxorubicin/peptide or doxorubicin alone to rats. As a result, it was hypothesized that the four peptides in this intestinal study would require stabilizers, unlike the nasal and in vitro studies of ZOT. Thus, levan, which is a fructose polymer with bioadhesive properties and is safely used in pharmaceutical applications, was used as a stabilizer in this study [23,24]. The study results demonstrated that levan could aid in the stabilization of Pep3 and Pep4, enabling the peptides to enhance the absorption of doxorubicin.

Unlike Pep3 and Pep4, Pep2 exhibited its intrinsic efficacy on the absorption of doxorubicin only in the presence of levan and BC, not levan alone, suggesting that each peptide requires its appropriate stabilizer. BC has been reported to contribute to the metabolic protection and stabilization of peptides [18]. Table 1 shows that neither levan alone nor with BC altered the absorbed amount or concentration of doxorubicin. Therefore, adding levan with BC is believed to have further increased the stabilization of Pep2.

The detailed peptide efficacy results showed that 10 mg·kg^−1^ of Pep1, Pep2, Pep3, and Pep4 in the presence of levan increased the AUC_0–240min_ of doxorubicin by 1.46-fold, 1.66-fold, 2.15-fold (*p* < 0.01), and 2.24-fold (*p* < 0.05), respectively, compared to the control solution of doxorubicin/levan (Table 1), and 10 mg·kg^−1^ of Pep2 with levan/BC increased the AUC_0–240min_ of doxorubicin by 2.08-fold (*p* < 0.05) compared to doxorubicin alone (Table 2). These results regarding additives are consistent with previous studies on FCIGRL and Pep1. When atenolol was administered intraduodenally to rats, Pep1 alone did not increase the AUC_0–360min_ of atenolol. However, adding levan to Pep1 increased the AUC_0–360min_ by 2.19-fold (*p* < 0.01) compared to atenolol alone [21]. In addition, FCIGRL, in the presence of protease inhibitors and BC, enhanced the AUC_0–120min_ of cyclosporin A (CsA) by 2.14-fold (*p* < 0.01) compared to CsA/protease inhibitors/BC [18].

When comparing the administration of doxorubicin by changing the concentration of the FCIGRL-modified peptides (Pep2, Pep3, and Pep4) at a constant concentration of levan or levan/BC, the AUC_0–240min_ of doxorubicin increased proportionally to the concentration of each peptide in the order of Pep4, Pep3, and Pep2.

FCIGRL is known to interact with proteinase-activated receptor-2 (PAR2) [25], which affects the structure and function of tight junctions with an accompanying increase in membrane permeability [26,27]. The four FCIGRL-modified peptides share carboxyl-terminal amino acid residues with the receptor binding site of ZOT and FCIGRL [15]. The results of this study indicate that Pep2, Pep3, and Pep4 have intrinsic permeation-enhancing properties that modulate tight junctions similar to ZOT.

Therefore, this study suggests that Pep2, Pep3, and primarily Pep4 with levan or levan/BC could be used as absorption enhancers for doxorubicin.

## 5. Conclusions

This study provides information on the effectiveness of FCIGRL-modified peptides as absorption enhancers in improving the intestinal membrane absorption of doxorubicin in rats. Peptides modified by cysteine dimerization and addition/substitution with N_3_-substituted dipropionic acid increased the absorption of doxorubicin. The addition of levan or levan/BC significantly affected the efficacy of the modified peptides. Therefore, Pep2, Pep3, and primarily Pep4 involved in tight junction opening represent novel absorption enhancers of significant importance in the delivery of drugs with low bioavailability due to membrane permeability, provided that they are stabilized by structural modification or formulation approaches.

## Figures and Tables

**Table 1:** Pharmacokinetic parameters in rats after the intraduodenal administration of doxorubicin (5 mg·kg^−1^) with each FCIGRL-modified peptide (10 mg·kg^−1^) and levan (0.5 *w*/*v*%).

	AUC_0–240min_ (min ng·mL^−1^)	C_max_ (ng·mL^−1^)	T_max_ (min)	T_1/2_ (min)	V_d_/F (L·kg^−1^)	Cl/F (mL kg·min^−1^)
Doxorubicin + levan + Pep4	9519.18 ± 953.95 * (2.24-fold)	198.99 ± 21.38 ** (2.97-fold)	5.00 ± 0.00 (0.71-fold)	226.57 ± 29.13 (0.89-fold)	112.25 ± 3.43 * (0.54-fold)	359.77 ± 63.88 (0.61-fold)
Doxorubicin + levan + Pep3	9144.32 ± 1343.45 ** (2.15-fold)	205.68 ± 25.84 ** (3.07-fold)	7.00 ± 1.22 (1.00-fold)	236.63 ± 22.15 (0.93-fold)	133.35 ± 17.61 * (0.64-fold)	390.52 ± 37.94 * (0.66-fold)
Doxorubicin + levan + Pep2	7084.95 ± 1459.19 (1.66-fold)	132.09 ± 19.61 * (1.97-fold)	11.67 ± 4.41 (1.67-fold)	243.63 ± 52.78 (0.96-fold)	184.98 ± 49.10 (0.89-fold)	514.63 ± 73.03 (0.87-fold)
Doxorubicin + levan + Pep1	6206.53 ± 1119.41 (1.46-fold)	156.94 ± 39.57 * (2.34-fold)	20.00 ± 0.00 ** (2.86-fold)	210.64 ± 10.91 (0.83-fold)	196.37 ± 39.05 (0.94-fold)	651.39 ± 137.14 (1.10-fold)
Doxorubicin + levan (control)	4258.49 ± 194.97	67.10 ± 12.03	7.00 ± 1.22	253.74 ± 30.70	208.20 ± 19.13	590.29 ± 64.15

Each data point represents the mean ± SEM of 3–5 rats. The values in the brackets indicate the fold-enhancement of each pharmacokinetic parameter compared to doxorubicin with levan (* *p* < 0.05; ** *p* < 0.01). Abbreviations: AUC, area under the curve; C_max_, peak concentration; T_max_, time to reach the peak; V_d_, volume of distribution; Cl, clearance; F, bioavailability.

**Table 2:** Pharmacokinetic parameters in rats after the intraduodenal administration of doxorubicin (5 mg·kg^−1^) and different concentrations of each FCIGRL-modified peptide, levan, and BC.

	AUC_0–240min_ (min ng·mL^−1^)	C_max_ (ng·mL^−1^)	T_max_ (min)	T_1/2_ (min)	V_d_/F (L·kg^−1^)	Cl/F (mL kg·min^−1^)
Doxo + levan (0.5 *w*/*v*%) + Pep4 (10 mg·kg^−1^)	9519.18 ± 953.95 ** (2.38-fold)	198.99 ± 21.38 ** (3.30-fold)	5.00 ± 0.00 (0.75-fold)	226.57 ± 29.13 (1.06-fold)	112.25 ± 3.43 * (0.52-fold)	359.77 ± 63.88 * (0.51-fold)
Doxo + levan (0.5 *w*/*v*%) + Pep4 (5 mg·kg^−1^)	8443.43 ± 1567.91 (2.11-fold)	147.34 ± 31.64 (2.44-fold)	10.00 ± 0.00 (1.50-fold)	232.67 ± 36.44 (1.09-fold)	152.42 ± 54.19 (0.70-fold)	442.77 ± 106.21 (0.63-fold)
Doxo + levan (0.25 *w*/*v*%) + Pep4 (10 mg·kg^−1^)	7225.12 ± 238.62 ** (1.81-fold)	104.06 ± 15.55 (1.72-fold)	10.00 ± 2.74 (1.50-fold)	266.83 ± 37.85 (1.25-fold)	130.94 ± 6.81 ** (0.60-fold)	359.30 ± 35.23 ** (0.51-fold)
Doxo + levan (0.5 *w*/*v*%) + Pep3 (10 mg·kg^−1^)	9144.32 ± 1343.45 * (2.29-fold)	205.68 ± 25.84 ** (3.41-fold)	7.00 ± 1.22 (1.05-fold)	236.63 ± 22.15 (1.11-fold)	133.35 ± 17.61 * (0.61-fold)	390.52 ± 37.94 ** (0.55-fold)
Doxo + levan (0.5 *w*/*v*%) + Pep3 (5 mg·kg^−1^)	7534.28 ± 1005.76 * (1.89-fold)	125.09 ± 17.40 * (2.07-fold)	8.00 ± 1.22 (1.20-fold)	251.33 ± 55.61 (1.17-fold)	127.69 ± 16.28 * (0.59-fold)	422.07 ± 103.89 (0.60-fold)
Doxo + levan (0.25 *w*/*v*%) + Pep3 (10 mg·kg^−1^)	6970.39 ± 2061.62 (1.75-fold)	190.03 ± 43.57 (3.15-fold)	6.67 ± 1.67 (1.00-fold)	185.51 ± 14.15 (0.87-fold)	139.90 ± 23.66 (0.64-fold)	536.14 ± 122.06 (0.76-fold)
Doxo + levan (0.25 *w*/*v*%) + Pep3 (5 mg·kg^−1^)	4400.23 ± 744.27 (1.10-fold)	119.66 ± 33.04 (1.98-fold)	5.00 ± 0.00 (0.75-fold)	269.95 ± 16.03 (1.26-fold)	254.94 ± 64.39 (1.17-fold)	668.18 ± 196.89 (0.95-fold)
Doxo + levan (0.5 *w*/*v*%) + BC (0.5 *w*/*v*%) + Pep2 (10 mg·kg^−1^)	8308.38 ± 979.26 * (2.08-fold)	134.20 ± 16.64 * (2.22-fold)	7.50 ± 1.44 (1.13-fold)	214.57 ± 28.83 (1.00-fold)	116.69 ± 12.28 ** (0.54-fold)	392.27 ± 53.17 ** (0.56-fold)
Doxo + levan (0.5 *w*/*v*%) + BC (0.5 *w*/*v*%) + Pep2 (5 mg·kg^−1^)	6723.07 ± 503.45 ** (1.68-fold)	92.85 ± 13.93 (1.54-fold)	9.00 ± 1.00 (1.35-fold)	231.27 ± 19.68 (1.08-fold)	137.78 ± 10.72 ** (0.63-fold)	422.87 ± 46.98 ** (0.60-fold)
Doxo + levan (0.5 *w*/*v*%) + Pep2 (10 mg·kg^−1^)	7084.95 ± 1459.19 (1.77-fold)	132.09 ± 19.61 * (2.19-fold)	11.67 ± 4.41 (1.75-fold)	243.63 ± 52.78 (1.14-fold)	184.98 ± 49.10 (0.85-fold)	514.63 ± 73.03 (0.73-fold)
Doxo + levan (0.5 *w*/*v*%) + Pep2 (5 mg·kg^−1^)	4090.05 ± 539.47 (1.02-fold)	115.95 ± 17.14 * (1.92-fold)	5.00 ± 0.00 (0.75-fold)	238.79 ± 5.21 (1.12-fold)	238.97 ± 29.87 (1.10-fold)	697.92 ± 103.17 (0.99-fold)
Doxo + levan (0.5 *w*/*v*%) + BC (0.5 *w*/*v*%)	3719.75 ± 554.33 (0.93-fold)	57.37 ± 15.70 (0.95-fold)	8.33 ± 1.67 (1.25-fold)	218.04 ± 38.81 (1.02-fold)	204.55 ± 12.93 (0.94-fold)	688.98 ± 120.39 (0.98-fold)
Doxo + levan (0.5 *w*/*v*%)	4258.49 ± 194.97 (1.07-fold)	67.10 ± 12.03 (1.11-fold)	7.00 ± 1.22 (1.05-fold)	253.74 ± 30.70 (1.19-fold)	208.20 ± 19.13 (0.96-fold)	590.29 ± 64.15 (0.84-fold)
Doxo alone (control)	3994.46 ± 326.83	60.33 ± 9.53	6.67 ± 1.67	214.02 ± 20.47	217.51 ± 20.98	704.36 ± 1.69

Each data point represents the mean ± SEM of 3–5 rats. The values in the brackets indicate the fold-enhancement of each pharmacokinetic parameter compared to doxorubicin alone (* *p* < 0.05; ** *p* < 0.01). Abbreviations: Doxo, doxorubicin; BC, benzalkonium chloride; AUC, area under the curve; C_max_, peak concentration; T_max_, time to reach the peak; V_d_, volume of distribution; Cl, clearance; F, bioavailability.

## Data Availability

Data are contained within the article.
